# Small versus Large Iron Oxide Magnetic Nanoparticles: Hyperthermia and Cell Uptake Properties

**DOI:** 10.3390/molecules21101357

**Published:** 2016-10-13

**Authors:** Cristian Iacovita, Adrian Florea, Roxana Dudric, Emoke Pall, Alin Iulian Moldovan, Romulus Tetean, Rares Stiufiuc, Constantin Mihai Lucaciu

**Affiliations:** 1 Department of Pharmaceutical Physics-Biophysics, Faculty of Pharmacy, “Iuliu Hatieganu” University of Medicine and Pharmacy, Pasteur 6, 400349 Cluj-Napoca, Romania; cristian.iacovita@umfcluj.ro; 2 Department of Cell and Molecular Biology, Faculty of Medicine, ‘‘Iuliu Hatieganu’’ University of Medicine and Pharmacy, Pasteur 6, 400349 Cluj-Napoca, Romania; aflorea@umfcluj.ro; 3 Faculty of Physics, “Babes Bolyai” University, Kogalniceanu 1, 400084 Cluj-Napoca, Romania; roxana.pacurariu@phys.ubbcluj.ro (R.D.); romulus.tetean@phys.ubbcluj.ro (R.T.); 4 Department of Reproduction Obstetrics and Veterinary Gynecology, University of Agricultural Sciences and Veterinary Medicine, Manastur 3-5, 400372 Cluj-Napoca, Romania; pallemoke@gmail.com; 5 Department of Bionanoscopy, MedFuture Research Center for Advance Medicine, “Iuliu Hatieganu” University of Medicine and Pharmacy, Pasteur 4-6, 400337 Cluj-Napoca, Romania; alin.moldovan@umfcluj.ro

**Keywords:** polyhedral iron oxide magnetic nanoparticles, large magnetic nanoparticles, polyethylene glycol, magnetic hyperthermia, specific absorption rate, cancer cells uptake, endocytosis, biodegradation

## Abstract

Efficient use of magnetic hyperthermia in clinical cancer treatment requires biocompatible magnetic nanoparticles (MNPs), with improved heating capabilities. Small (~34 nm) and large (~270 nm) Fe_3_O_4_-MNPs were synthesized by means of a polyol method in polyethylene-glycol (PEG) and ethylene-glycol (EG), respectively. They were systematically investigated by means of X-ray diffraction, transmission electron microscopy and vibration sample magnetometry. Hyperthermia measurements showed that Specific Absorption Rate (SAR) dependence on the external alternating magnetic field amplitude (up to 65 kA/m, 355 kHz) presented a sigmoidal shape, with remarkable SAR saturation values of ~1400 W/g_MNP_ for the small monocrystalline MNPs and only 400 W/g_MNP_ for the large polycrystalline MNPs, in water. SAR values were slightly reduced in cell culture media, but decreased one order of magnitude in highly viscous PEG1000. Toxicity assays performed on four cell lines revealed almost no toxicity for the small MNPs and a very small level of toxicity for the large MNPs, up to a concentration of 0.2 mg/mL. Cellular uptake experiments revealed that both MNPs penetrated the cells through endocytosis, in a time dependent manner and escaped the endosomes with a faster kinetics for large MNPs. Biodegradation of large MNPs inside cells involved an all-or-nothing mechanism.

## 1. Introduction

Magnetic nanoparticles (MNPs) represent one of the main classes of nanoparticles (NPs) which are currently in the research spotlight, with many potential applications in the field of the life sciences: magnetic resonance imaging contrast enhancement, tissue repair, immunoassay, detoxification of biological fluids, drug delivery, hyperthermia, and cell separation [1]. The scientific literature reports the use of MNPs in routine laboratory and clinical protocols, such as cell sorting, DNA separation, magnetic resonance imaging (MRI) and gene therapy [2]. Applications of MNPs currently being in preclinical stages include cell-targeted delivery of anticancer agents and molecular diagnosis [3]. Starting with the pioneering work of Gilchrist et al. in 1957 [4], MNPs have also been proposed for hyperthermia applications, preferentially for cancer therapy. This new therapeutic concept, called *magnetic hyperthermia* (MH) [5], relies on the heat released by the MNPs exposed to an externally applied alternating magnetic field (AMF) which is used to increase the temperature of the cancer cells, up to a level at which apoptosis can be initiated. However, as Gilchrist et al. [4] initially pointed out in their seminal work, the efficient use of magnetic hyperthermia in clinical cancer treatment clearly demands MNPs with highly improved properties: (i) they should be able to deliver sufficient heat to completely destroy the tumor at the lowest particles dose and at safety levels of AMF; (ii) they must be biocompatible to easily circulate through the blood stream and (iii) they should be able to specifically target the cancer cells and to reliably provide a controlled intratumoral heat exposure [6,7].

The heating capabilities of MNPs under external AC magnetic field are quantified by the specific absorption rate (SAR) parameter (some authors use the term specific loss power (SLP) to describe the same physical reality), which provides a measure of the rate at which energy is absorbed per unit mass of the magnetic nanoparticles [8]. SAR values depend on MNP’s structure, size, size distribution, shape and composition but also on the frequency (f) and the amplitude of the external AC magnetic field (H) applied [9]. For clinical applications, it is largely accepted that the product between the frequency (f) and the magnetic field amplitude (H), tolerable for patients, should be smaller than 5 × 10^9^ Am^−1^s^−1^ [10], owing to the fact that both, high frequency and high amplitude AMFs produce eddy currents in conducting media.

Among all types of magnetic nanoparticles (MNPs) developed so far, Fe_3_O_4_ superparamagnetic iron oxide nanoparticles (SPION) are the only type of MNPs approved for clinical use by the US Food and Drug Administration [11]. They have already been tested in vivo for clinical MH therapy [12] due to their excellent biocompatibility and stability. This form of therapy was clinically approved in Europe for treatments of glioblastoma in 2011 and a clinical trial was also performed on prostate cancer [12]. However, it has been shown that the SAR values of spherical SPIONs with different diameters are relatively low (a few hundred W/g), and their heating power drastically decreases when the MNPs are localized into cells or tissues [13]. Consequently, in order to facilitate the complete elimination of the tumor, the magnetic hyperthermia has been used in conjunction with classical therapies (chemo and/or radio therapies), but in this case aggressive sides effects, have been observed. These limitations can be overcome by using higher SPION doses in conjunction with higher AC magnetic fields amplitude and higher frequencies, but in this case the safety value H × f factor is exceeded and various side effects can also appear. As a result, lowering the dose levels, below their intrinsic toxicity levels and enhancing the heating capabilities of MNPs have become the major goals for the scientific community involved in magnetic hyperthermia research.

Several theoretical models were developed in order to understand the basic mechanisms and to identify the main parameters that govern the MNPs heat dissipation process in AMF. For SPIONs the main processes governing the heat dissipation is related to Neel and Brown relaxation processes, which are described by the so-called Linear Response Theory, developed by Rosensweig [8]. For larger MNPs, in the ferromagnetic domain, theories derived from the Stoner and Wohlfarth model [14] were developed [15]. These theories underscore the importance of the hysteretic characteristics of the MNPs (coercive field, saturation magnetization) and of the amplitude of the external AMF. In the case of an intermediate regime, none of the above models can be applied and numerical methods are usually employed.

The scientific literature is rich in experimental studies related to the heating capabilities of different SPIONs and other classes of MNPs prepared by various synthesis methods, leading to some general observations: small (~10 nm) SPIONs have a limited SAR value of a few hundreds of W/g; larger MNPs, in the ferromagnetic domain exhibit SAR value with one order of magnitude higher than SPIONs; above a size threshold value, the MNPs might enter into a multi magnetic domains state, leading to a decrease in their SAR values; cubic [16], nanorods [17] and octahedral [18] MNPs display outstanding SAR values relative to their spherical counterparts.

The MNPs exhibiting high SAR values were mainly synthesized by means of non-hydrolytic thermal decomposition methods. Besides the inconvenient that MNPs are not water-dispersible, this method can lead to MNPs formed by mixed Fe_3_O_4_ magnetite and FeO wüstite phases with low saturation magnetization and SAR values. Usually they require a post-synthesis treatment in order to improve their magnetic properties and for rendering them both hydrophilic and biocompatible [19]. Moreover, larger size MNPs exhibiting higher SAR values need a seeded growth mediated synthesis which can lead to polycrystalline structures and can compromise their heating capabilities. Recently, we have extended the polyol based synthesis method for the synthesis of cubic and polyhedral Fe_3_O_4_ MNPs by using polyethylene glycol (PEG) as both solvent, and capping agent [20]. These MNPs exhibit excellent crystallinity and SAR values (~1600 W/g_MNP_ at f = 355 kHz and H = 60 kA/m) higher than SAR values given by the SAR values observed for spherical MNPs and comparable with those given by the non-spherical MNPs synthesized using non-hydrolytic methods. The polyol based synthesis method offers several advantages over other methods as it produces MNPs with good crystallinity, in a one step process, without the need of hydrophilization and can be easily scaled for producing large amounts of MNPs. Moreover, this method has been largely employed to synthesize biocompatible MNPs of variable sizes and morphologies, either by the choice of the solvent, or by finely tuning the duration of the reaction and the concentration of magnetic precursor.

On the other hand, for a proper use of MNPs in biomedical applications it is important to evaluate their toxicity. Their potential impact on human health must be thoroughly assessed and information concerning the risks to be avoided, such as inflammatory, genotoxic, oxidative, or cytotoxic effects, should be provided [21]. Many studies were conducted in cell cultures for assessing the cytotoxicity of MNPs and their uptake mechanisms [22]. The internalization of nanomaterials by living systems raises big concerns; due to their small size, they have the ability to penetrate biological membranes, they can reach the cells nuclei and deregulate signaling pathways. They can also affect cell morphology and cytoskeletal networks, hence causing cellular deregulation, toxicity, and cell death. The results showed that the MNPs toxic levels depend on the size, nature, shape and coating of the MNPs and also on the cell line types they were tested on [23]. In order to address these challenges, complete toxicology studies are mandatory in order to understand the impact of the exposure of cell cultures to MNPs [24].

Based on these experimental observations, in the present study we propose to systematically investigate and compare the structural, magnetic, hyperthermic, cytotoxicity and cell uptake properties of two classes of MNPs: Fe_3_O_4_ MNPs synthesized in PEG 200 and ethylene glycol (EG), called in the followings small and large Fe_3_O_4_ MNPs, respectively. PEG 200 favors the formation of polyhedral Fe_3_O_4_ MNPs with an average size of ~34 nm, whereas at similar reaction times and at the same molar ratio between solvent and magnetic precursor, EG produces large spherical Fe_3_O_4_ MNPs with an average size of ~270 nm, as previously demonstrated by Deng et al. [25]. XRD studies showed that both samples consist of pure magnetite, with almost similar crystallite size, while the magnetic properties at room temperature are similar as well. Despite this similitude, the two types of MNPs display completely different hyperthermia properties. Since it has been demonstrated that SAR values of MNPs are temperature-dependent [26] and both the in vivo and in vitro magnetic hyperthermia applications require a starting temperature of 37 °C, the hyperthermia performance of MNPs has been evaluated in an environment held at 37 °C and compared with that obtained at the room temperature (24 °C–25 °C). Several different media (water, cell culture media and PEG1000 in both liquid and solid state), have been used for a complete comprehension of hyperthermic properties of both types of MNPs.

In order to have a better picture of their cytotoxicity and cellular uptake, the interaction of MNPs with four different cells lines: human retinal pigment epithelial cells (D407), human lung carcinoma cells (A548), human melanoma cells (MV35) and mouse melanoma cells (B12F10) have been considered. The MNPs cytotoxicity has been assessed by using the standard 3-(4,5-dimethylthiazol-2-yl)-2,5-diphenyltetrazolium bromide (MTT) assay up to a concentration of 0.2 mg/mL, while their uptake by the cells was monitored by Transmission Electron Microscopy (TEM).

## 2. Results and Discussion

### 2.1. Structural Characterisation

**Figure 1:**
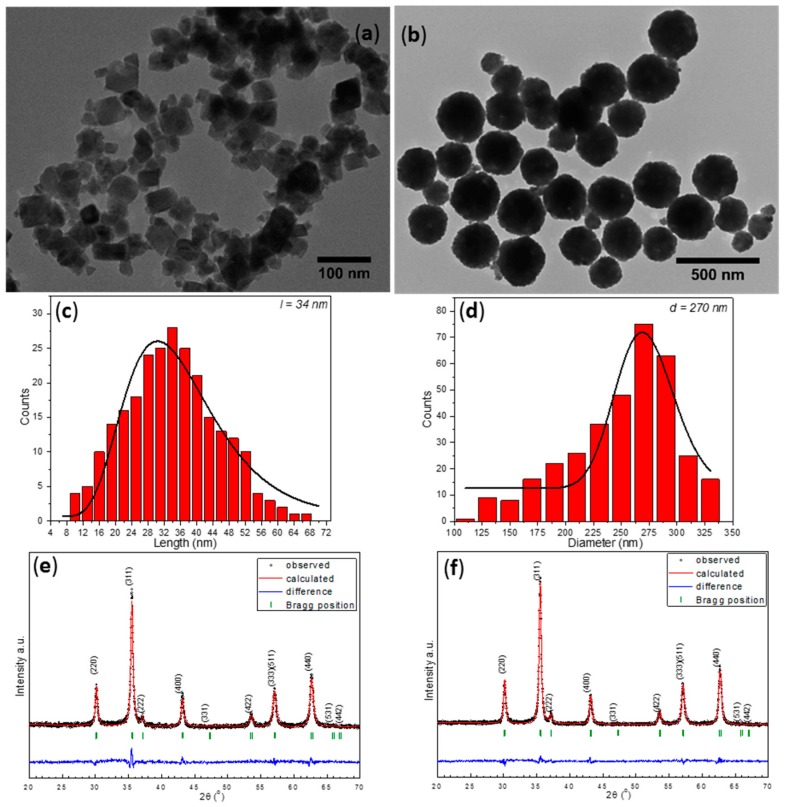
TEM images of Fe_3_O_4_ MNPs synthesized in (**a**) PEG 200; and (**b**) EG and their corresponding size distribution histograms (**c**,**d**) fitted to a log-normal distribution (black lines) and XRD diffractions patterns (**e**,**f**).

As a first step, TEM and XRD techniques were used to analyze the shape and the structure of MNPs. In Figure 1a,b, the TEM images of both type of MNPs are presented. A molar ratio of 1:270 between PEG200 and iron chloride kept at 240 °C for 6 h favored the formation of polyhedral MNPs, with a relatively broad size distribution and an average size of 34 nm (Figure 1a,c). Different shapes can be distinguished in the TEM images: cubic, parallelepipedic, octahedral. Large spherical MNPs with a mean diameter of 270 nm were formed in EG at 200 °C for 6 h (Figure 1b,d), by keeping the same molar ratio of 1:270 between EG and iron chloride.

To further analyze the crystalline structure of the MNPs, X-ray diffraction (XRD) was performed on powder samples that obtained after drying MNPs suspension in a rota-evaporator. As shown in Figure 1e,f, the XRD pattern clearly revealed the existence of a pure inverse spinel crystalline structure in both samples. The position and the relative intensities of all diffraction peaks ascribed to magnetite Fe_3_O_4_ (PDF number: 88-0315 [27]). No FeO or Fe_2_O_3_ peaks were found in the XRD pattern, indicating that all MNPs consist of pure magnetite Fe_3_O_4_. The black color of the powder was a further confirmation that the samples were of pure magnetite phase. The corresponding lattice parameters (a = 8.383 Å and a = 8.379 Å for Fe_3_O_4_ synthesized in PEG200 and EG respectively) were very close to that of bulk magnetite (a = 8.375 Å). The crystalline size of Fe_3_O_4_ synthesized in PEG200, calculated from the (311) diffraction peak using Debye-Scherrer’s formula was 28.5 nm, which is smaller than the average length obtained from TEM images. At this point we would like to notice that the size revealed by the XRD data corresponds with the smallest crystallites, as these crystallites are those which give the largest broadening in the XRD peaks. The relatively broad size distribution of MNPs suggests that most of the Fe_3_O_4_ MNPs are single crystals. Surprisingly, a similar crystalline diameter of 27.3 nm measured by XRD, has been found for Fe_3_O_4_ synthesized in EG. The large difference between the XRD data and those from TEM clearly suggested that the large spherical Fe_3_O_4_ MNPs displayed a polycrystalline structure and were constituted of multiple crystals.

In order to characterize the MNPs suspensions we performed Dynamic Light Scattering (DLS) measurements at different concentrations (Figure S1a,b). The results showed that the hydrodynamic dimensions provided by the DLS data are strongly dependent on suspension concentration.

For large MNPs, at relatively high suspension concentrations (~1 mg/mL) similar to those used in hyperthermia experiments, the mean hydrodynamic diameter was ~1100 nm (Figure S1b). This value decreases as the concentration of MNPs decreased. For very diluted suspensions (less than 5 μg/mL), the hydrodynamic diameter is around 250–300 nm which is well correlated to the mean diameter obtained from TEM data. These results suggest that at high concentration a concentration dependent agglomeration of the MNPs can be envisaged.

Small polyhedral MNP, at a concentration of 1 mg/mL exhibited a hydrodynamic diameter around 325 nm, which decreased slowly by increasing the dilution (Figure S1a). However, starting at a concentration of 0.2 mg/mL DLS data revealed the appearance of a second maximum at ~70 nm. Continuing the dilution up to a concentration of 1 μg/mL the size distribution revealed a single maximum at a value of ~130 nm, almost four times larger than the dimensions measured by TEM. This behavior suggests that the small polyhedral MNPs, even in very dilute samples, aggregate in small clusters, comprising several individual MNPs and, as the concentration increases, the interaction between the individual aggregates may lead to the formation of larger agglomerated structures.

One can conclude that both types of MNPs are in an aggregated/agglomerated form at concentrations where hyperthermia measurements were performed. While the large MNPs suspensions can be dispersed, in very dilute samples, up to a level at which their size is similar to that obtained from TEM measurements (~270 nm), the small MNPs remain organized in aggregates with a mean diameter of ~130 nm, even for the most diluted samples.

### 2.2. Magnetic Properties

**Figure 2:**
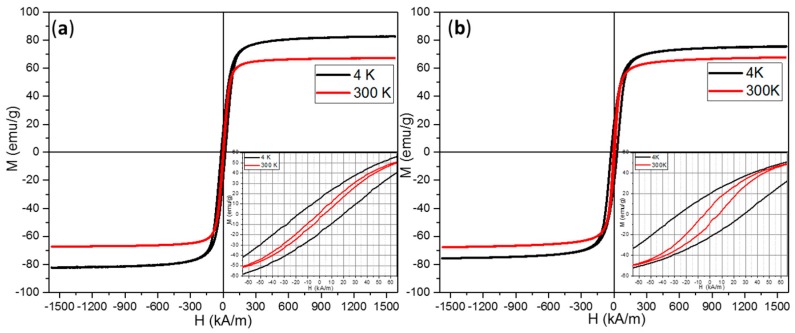
Magnetic hysteresis loops of Fe_3_O_4_ MNPs synthesized in (**a**) PEG 200; and (**b**) EG. The insets are a zoom-in the hysteresis loops.

The magnetic properties of Fe_3_O_4_ MNPs (Table 1) were measured using a vibrating sample magnetometer (VSM). At low temperature (4 K), both types of Fe_3_O_4_ MNPs showed hysteresis curves with similar shapes (Figure 2—black lines), revealing the standard ferromagnetic character exhibited by iron oxide MNPs. The Fe_3_O_4_ MNPs synthesized in PEG200 displayed a saturation magnetization (M_s_) of 82 emu/g, which is slightly higher than the M_s_ of Fe_3_O_4_ MNPs synthesized in EG (75 emu/g). Large MNPs are expected to display M_s_ close to the value obtained for the bulk magnetite (92 emu/g). In our case, the large magnetic core of the Fe_3_O_4_ MNPs synthesized in EG was formed by aggregation of multiple crystallites, which could possess cation vacancies (arising from the synthesis) and thus reducing the M_s_. Since the majority of the smaller Fe_3_O_4_ MNPs synthesized in PEG are constituted mainly from individual crystals, the amount of cation vacancies was reduced and hence the M_s_ had higher values. On the contrary, the large Fe_3_O_4_ MNPs had higher coercive field (H_c_) and remanence (M_r_) values at 4 K (Table 1), probably due to the magnetic interactions that occur between crystallites within the magnetic core. The M_r_/M_s_ ratios were smaller than 0.5 indicating an uniaxial anisotropy. The anisotropy constants were calculated as K_eff_ = μ_0_H_c_M_s_/0.96 [14]. The effective anisotropy at low temperatures was very close to the bulk magnetite values (11–13 kJ/m^3^) with slightly higher values for the large Fe_3_O_4_ MNPs, indicating the good quality of our Fe_3_O_4_ MNPs. The differences between the effective anisotropy constants of the two types of Fe_3_O_4_ MNPs were much larger at 300 K, once again the value being bigger for large Fe_3_O_4_ MNPs. We recall that the XRD data suggested that the two sets of MNPs consisted of crystallites with almost identical sizes. The relatively large values of the characteristic size of these crystallites (~30 nm) reduces the contribution of the surface anisotropy to the effective anisotropy for both types of Fe_3_O_4_ MNPs. The differences in the K_eff_ can be attributed mainly to the shape anisotropy. The small Fe_3_O_4_ MNPs had cubic, octahedral and polyhedral shapes with lower index facets, leading to a reduced contribution of the surface to the overall anisotropy constant as pointed out by Noh et al. [28].

Hysteresis loops, recorded at room temperature (Figure 2a,b, red lines), indicated a reduction of all magnetic parameters (Table 1). Independent on the size of MNPs, the M_s_ considerably dropped to a value of 68 emu/g (Table 1), which was similar to M_s_ reported for octahedral magnetite of the same size [18].

Even though the H_c_ and remanence (M_r_) values at room temperature of small and large Fe_3_O_4_ MNPs were very small (Table 1), their behavior was not superparamagnetic at room temperature. This can be clearly seen in the zero-field-cooled/field-cooled (ZFC/FC) magnetization curves (Figure S2) that start to join at 300 K. The maximum in ZFC curves, which corresponds to the average onset of the ferromagnetic to superparamagnetic transition, is located at the same temperature. This maximum is broadened, suggesting that the gradual transition to the superparamagnetic state is extended above 300 K, all MNPs being in a superparamagnetic state at temperatures well above room temperature. Thus, the Fe_3_O_4_ MNPs are in a ferromagnetic state at room temperature and above (i.e., the temperature range where hyperthermia experiments were conducted). In addition, the ZFC magnetization curves showed a change of magnetization at ~ 50 K, which might be related to the structural transition from high temperature cubic to low temperature monoclinic structure [29]. The signature of the thermally activated first-order Vervey transition around ~100 K [30] was faintly visible. The M(T) values of FC curves can provide information about the inter-particle interaction strength [31]. For small Fe_3_O_4_ MNPs, the average M(T) value was 37 emu/g, while in the case of large Fe_3_O_4_ MNPs this value was 24 emu/g. This observation suggests that large Fe_3_O_4_ MNPs would exhibit stronger inter-particle interactions as compared to small Fe_3_O_4_ MNPs that can induce a variation of the SAR values.

### 2.3. Hyperthermia Properties

As demonstrated above, at room temperature the MNPs are in a blocked state and can develop attractive interparticle interactions that favor the formation of aggregates when they are dispersed in water. In order to ensure colloidal stability and to hamper the formation of big clusters, the MNPs were treated with tetramethylammonium hydroxide (TMAOH), as presented in our previous study [20]. The heat performance of MNPs was measured in an environment held either at room temperature or at 37 °C. For a better characterization of SAR evolution with the magnetic field amplitude, the latter was varied between 5 kA/m and 65 kA/m in steps of 5 kA/m. The frequency of the oscillating magnetic field was steered to 355 kHz, the highest value in our set-up. A reliable SAR measurement requires a uniform distribution of the MNPs in the sample volume. Therefore, prior to each set of SAR measurements the samples were sonicated for 30 s. As briefly described in Supplementary Materials, the SAR values were calculated by measuring the initial slope of temperature vs. time curves and normalized to the Fe_3_O_4_ MNPs amount, considering the heat capacity of medium.

**Figure 3:**
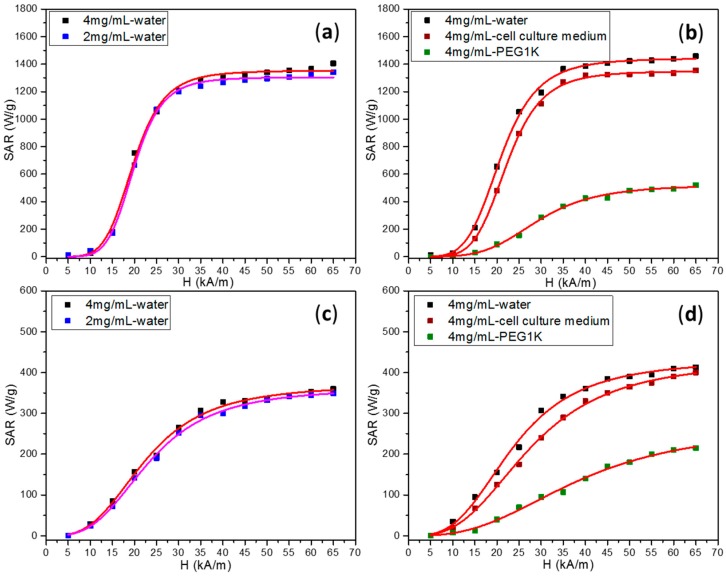
SAR values of Fe_3_O_4_ MNPs synthesized in PEG 200 starting at 25 °C (**a**) and at 37 °C (**b**); and EG starting at 25 °C (**c**) and at 37 °C (**d**) as a function of applied AC magnetic field amplitude and at a frequency of 355 kHz. The data were fitted with a sigmoidal function (Equation (3)) (red and pink lines).

The characteristic heating curves of Fe_3_O_4_ MNPs dispersed in water and different other media (Figures S3 and S4), exhibit a considerable temperature increase, upon applying the AC magnetic field. This allowed the hyperthermia treatment to reach the therapeutic temperature (45 °C) in a time span ranging from several seconds to a few min. The time needed for the temperature to reach 45 °C is a function of AC magnetic field amplitude, the dispersing medium, as well as the size and concentration of the Fe_3_O_4_ MNPs. The dependence of the SAR values of both types of Fe_3_O_4_ MNPs as a function of the AC field amplitude was a sigmoidal one, clearly revealing the saturation of SAR values for high fields (Figure 3).

In fact, for large MNPs in the ferromagnetic regime, the linear response theory, developed by Roseinsweig [8], is not valid anymore and models describing the hyperthermic behavior are based on the Stoner and Wohlfarth theory [14]. These authors considered the limit of anisotropic ellipsoids possessing only two orientations possible for the magnetization, without taking into account a thermal activation (T = 0). The magnetization can be reversed only by magnetic fields above a critical value H_k_. The hysteresis loop is rectangular and the coercive field equals the critical field and the anisotropy field. The area of the hysteresis loop is maximum and gives us the upper limit of the SAR for a given material:

(1) $$SAR=P/\rho =Af/\rho =\;4\mu_{0}M_{s}H_{c}f/\rho$$

where A stands for the area of the hysteresis loop, M_s_ is the saturation magnetization, H_c_ is the coercive field and ρ is the density. According to this theory, for magnetite with M_s_ = 480 kA/m and H_c_ = 30 kA/m the maximum achievable SAR at 500 kHz is 7 kW/g.

However, in the real case of randomly oriented MNPs, the coercive field is reduced to 0.48 from the critical field and, as a consequence, the maximum SAR is reduced in the same field conditions to about 1/2 from the pure Stoner-Wohlfarth NPs [15]. For magnetic field amplitudes smaller than the coercive one, unable to reverse the magnetization of the MNPs, no energy absorption takes place. Therefore this model holds true only at AC magnetic field amplitudes surpassing the coercive field, when the hysteresis loop is a major one and the MNPs are saturated by the magnetic field. The SAR dependence on the AC magnetic field amplitude is sigmoidal showing a saturation at high AC magnetic field amplitude values. Carrey et al. [15] and Mehdaoui et al. [32] showed that in the case of high AC magnetic field amplitudes the coercive field can be obtained from hyperthermia experiments (H_cHyp_), and its value can lead to the optimal size of MNPs maximizing the SAR. The H_cHyp_ represents the point of highest slope of the SAR = f(H) curve and is calculated from the equation:

(2) $$\mu_{0}H_{cHyp}=0.463\mu_{0}H_{k}\{1-{\left[\frac{k_{B}T}{KV}ln\left(\frac{k_{B}T}{4\mu_{0}H_{cHyp}M_{s}Vf\tau_{0}}\right)\right]}^{0.8}\}$$

where H_k_ represents the critical field, f is the frequency, $\tau_{0}$ is the attempt time while the other letters have their usual physical meaning . The results obtained for Fe MNPs [32] for the calculated coercive fields and the experimental SAR were in good agreement, the SAR = f(H) curves having a sigmoidal shape. 

As it can be observed in Figure 3, our experimental SAR = f(H) curves have also sigmoidal shapes reaching saturation at about twice the coercive field (Table 2). Our experimental data were well fitted (R^2^ > 0.999) phenomenologically with a simple logistic function:

(3) $$SAR=SAR_{max}\frac{{\left(\frac{H}{H_{cHyp}}\right)}^{n}*\propto }{1+{\left(\frac{H}{H_{cHyp}}\right)}^{n}*\propto }$$

with:

(4) $$\propto =\frac{n+1}{n-1}$$

were SAR_max_ represents the saturation value of the SAR, H_cHyp_ is the hyperthermic coercive field, the value of the AC magnetic field amplitude for which the function presents the higher slope [15] and the exponent *n* indicates how steep the dependence of SAR on the amplitude of the AC magnetic field is. Numeric first-order derivation of the SAR = f(H) curves are provided in Figure S6, showing the AC magnetic field amplitude value corresponding to the maximum slope.

As can be seen in the Figure 3a,c, where the data for both types of Fe_3_O_4_ MNPs suspended in water at two concentrations were represented, at very low AC fields (5 kA/m and 10 kA/m) the SAR values are extremely small. The Fe_3_O_4_ MNPs synthesized in PEG200 started to deliver a considerable amount of heat (~180 W/g) once the AC field was increased at 15 kA/m (Figure 3a). At 20 kA/m, the SAR values abruptly increase to ~750 W/g, while for an AC field of 25 kA/m the attained SAR was almost 1050 W/g. By further increasing the AC fields from 30 kA/m to 40 kA/m, the SAR values gradually increased to ~1315 W/g. For AC fields higher than 40 kA/m the SAR values reached a saturation of ~1350 W/g. As depicted in Figure 3c, the heating behavior of large Fe_3_O_4_ MNPs followed the same trend as a function of AC fields, but the SAR values were much lower. In the 15 kA/m–40 kA/m AC fields range, the SAR values gradually increased from 85 W/g up to 330 W/g, whereas for AC fields higher than 40 kA/m the SAR values increased smoothly to a saturation value of 370 W/g.

These results agree qualitatively with the numerical simulation performed by Carrey et al. [15] and Christiansen et al. [33]. Indeed, for large ferromagnetic nanoparticles, the numerical simulation revealed a steep transition as a function of the AC magnetic field, from a regime where the hysteresis area is very small to a regime where the hysteresis area is very large. The explanation for this behavior is related to the coercive field (H_c_). When the applied AC magnetic field (H) is smaller than H_c_ the hysteresis area is very small, while when H is larger than H_c_ the hysteresis area is larger. On the other hand, for very small AC magnetic fields the SAR dependence can be described by a power law. The main difference in the two sets of data is the value of the saturation magnetization which is higher for the small Fe_3_O_4_ MNPs. However, we were not able to find a clear-cut correlation between the maximum SAR values and the differences measured in the magnetic properties of the two sets of Fe_3_O_4_ MNPs. As revealed from the TEM and XRD data, the larger Fe_3_O_4_ MNPs were polycrystalline while the small Fe_3_O_4_ MNPs were mostly single crystals. Therefore, the dipolar magnetic interaction between the crystals within the large Fe_3_O_4_ MNPs may lead to a strong decrease in the SAR values. Our results are consistent with recent reports on hyperthermic behavior of three classes of MNPs with different magnetic core structures [33]. The mentioned study demonstrates that parallelepipedic ferrite nanoparticles (obtained at high temperatures and pressures) with a mean TEM size of 20 nm have SAR values 3–4 times higher than MNPs composed of multiple crystallites embedded in a dextran matrix, of the same size (measured by TEM), although their magnetic properties are similar. Moreover, their SAR = f(H) is also sigmoidal, saturating at high AC magnetic fields amplitudes. The main reason for the different heating capabilities is related to their different magnetic domain structures, as it was revealed by small angle neutron scattering data [34].

The hyperthermia coercive fields (Table 2) were very close to the coercive fields measured in static conditions, at low temperatures (Table 1) ~20 kA/m for small Fe_3_O_4_ MNPs. For large Fe_3_O_4_ MNPs the H_cHyp_ were smaller as compared to their static value (H_cHyp_ = 18 kA/m and H_c_ at 4 K is 27 kA/m). These results suggest that the hyperthermic coercive field (the point of highest slope in the SAR = f(H) function) is strongly related to the intrinsic magnetic properties of the Fe_3_O_4_ MNPs, but is also influenced by the interactions between MNPs and, as it is presented below, by their environment. Another important feature in the parameters derived from the SAR = f(H) dependencies is the power coefficient n (exponent) in Equation (3). As one can easily see, for water and cell culture media, in the case of small Fe_3_O_4_ MNPs, the n value was in the range of 5–6, while for large Fe_3_O_4_ MNPs the values of n were almost halved. This indicates a much steeper dependence of SAR on the applied AC magnetic field in the case of small Fe_3_O_4_ MNPs and a better suitability of Stoner-Wohlfart derived models for the description of their hyperthermic behavior.

The SAR values for each AC field amplitude given by both samples with a concentration of 2 mg/mL of Fe_3_O_4_ MNPs were almost similar with those previously obtained on a more concentrated sample (Figure 3a,c). Although the Fe_3_O_4_ MNPs were in a ferromagnetic state at room temperature, the interparticle dipole-dipole interactions developed by Fe_3_O_4_ MNPs were expected to influence the SAR values in such a way that by decreasing the concentration of Fe_3_O_4_ MNPs their SAR will be improved. Many papers reported apparently contradictory data on the SAR dependence on the concentration of MNPs, some of them being summarized in references [35,36].

We believe that the hyperthermic properties should be discussed also in connection with the DLS data. The aggregation/agglomeration influences the hyperthermic properties of the MNP, that depend both on the dimensions of individual nanoparticles and on the hydrodynamic diameter of the aggregates, as pointed out recently by Deatsch and Evans [36] and Blanco-Andujar et al. [37].

Deatsch and Evans explained the decrease in SAR with the increase in MNPs concentration based on the dipole-dipole interaction, leading to chain formation [36]. They interpreted this effect by making a distinction between aggregation and agglomeration and also between the two relaxation mechanisms, Brown and Neel. Recently Bianco-Andujar et al. [37] reported that the decrease in the heating performance of MNPs was due to the demagnetizing effect of the interparticle interactions. They also observed that multicore nanoparticle aggregates exhibit higher SAR values when larger core nanoparticles aggregate in smaller complexes than in the opposite situation. In our particular case we believe that for the large Fe_3_O_4_ MNPs the lower SAR values as compared to the small Fe_3_O_4_ MNPs can be attributed to magnetic interactions between the magnetic crystallite aggregates. Moreover, the similar SAR values obtained for different concentrations indicate that the inter-aggregates interactions are rather weak and consequently they do not affect the SAR (Table 2).

As seen in the DLS data, up to a dilution of 0.4 mg/mL, for both types of MNPs, there is no significant change in the aggregate dimensions which were ~325 nm for small MNPs and 1100 nm for large MNPs, respectively. Therefore, the SAR data do not differ significantly in the 2–4 mg/mL concentration range. One can expect a change of the SAR at concentrations with at least one order of magnitude lower, at which we noticed significant changes in the dimension distribution profiles for both types of MNPs. However, as our set up is not adiabatic, for these very low concentrations, significant errors can occur.

In order to establish the reliability of Fe_3_O_4_ MNPs as potential heating mediators for magnetic hyperthermia cancer treatment, it is of utmost importance to evaluate their hyperthermia properties in an environment close to in vivo and in vitro characteristics. In this regard the Fe_3_O_4_ MNPs were dispersed in three different media, displaying different viscosities: water, cell culture medium and PEG1000.The environment inside the coil was held at 37 °C, this temperature being also the starting temperature in the hyperthermia experiments. It was found out that the hyperthermia performance of both types of MNPs, in water, slightly increased for both samples (Figure 3b,d). For a starting temperature of 37 °C, the SAR values of MNPs and its evolution as a function of the AC field amplitude (Figure 3b,d) was almost similar with that acquired starting at 25 °C (Figure 3a,c). The fittings highlight an increase in the saturation values of SAR to 1442 W/g and 431 W/g for small and large MNPs, respectively. These results are consistent with a recent report from Garaio et al. [26], that showed by using dynamic hysteresis (AC magnetometry), that for small MNPs (less than 16 nm) SAR decreased with increasing temperature, while for larger Fe_3_O_4_ MNPs, SAR increased with increasing temperature. 

A slight reduction of maximum SAR values of both types of Fe_3_O_4_ MNPs, without changing its sigmoidal shape nor the H_cHyp_ was recorded in cell culture medium (Figure 3b,c). This behavior suggests that Fe_3_O_4_ MNPs were not significantly affected by the physiological conditions, as different components of the cell culture medium may attach to the Fe_3_O_4_ MNPs surface and induce aggregation, thus potentially being able to affect the heating performance. 

A significant drop of SAR values was observed when both types of MNPs were dispersed in PEG1000 (Figure 3b,d and Figure S5). Up to 37 °C PEG 1000 is a soft solid and after melting at 37 °C it becomes a, highly viscous liquid, mimicking the cellular medium. Therefore, at the melting point we expect to have a sudden change in the hyperthermic properties as MNPs pass from an immobilized state (in the solid PEG matrix) to a mobile state in the liquid PEG. Indeed, this change in the thermal behavior of the MNPs can be clearly seen in the Figures S3c and S4c, where the temperature change versus time curves were recorded. Up to ~37 °C, the MNPs display a low SAR value, reflected in a smaller slope of the T = f (time) curve, while above 37 °C, when PEG1000 melts, their mobility is increased and their SAR and the slope of the T = f (time) curve increase also. Owing to this dual behavior, we calculated the SAR vs. magnetic field strength curves for slopes bellow 37 °C, (Figure S5) and above 37 °C (Figure 3b,d, green squares). The calculated SAR vs. H parameters for the two situations are given in Table 2.

As one can easily notice there is a strong decrease in the SAR values of both types of Fe_3_O_4_ MNPs when analyzed in either solid or liquid phase of PEG 1000, however these decreases being much more pronounced for the case when the PEG 1000 is a soft solid. The sigmoidal shape of the SAR curves is less pronounced as the power coefficients drop to ~2 and the H_cHyp_ increases ~1.5 times for both types of Fe_3_O MNPs (Figure S5 and Table 2). According to the fittings, the SAR saturation values for small and large MNPs, dispersed in PEG1000 being in liquid state, decreased to ~ 520 W/g (64% drop) and ~270 W/g (38% drop), respectively. For soft solid PEG 1000 the maximum SAR values, calculated for the slopes between 25 °C and 37 °C were much smaller, 179 W/g (87.5% drop) and 95 W/g (78% drop) for small and large MNPs, respectively. We recall that our Fe_3_O_4_ MNPs were in a blocked state above room temperature. Therefore, the heating mechanism would be mainly based on hysteresis losses when the PEG is in a soft solid state and on hysteresis losses and Brownian friction when PEG 1000 is liquid. Once dispersed in soft solid PEG1000, the Brownian contribution was suppressed and the magnetic anisotropy losses were the major contributor to power dissipation. In these conditions, the evolution of SAR values could be proportional to the increase of the hysteresis loop area with the AC field amplitude. A closer inspection of the hysteresis loops in the 0–65 kA/m range (insets of Figure 2a,b) evidence a thinner hysteresis area of small Fe_3_O_4_ MNPs compared with large Fe_3_O_4_ MNPs, suggesting that the latter should deliver more heat. This is not the case, the small Fe_3_O_4_ MNPs immersed in PEG1000 displayed double SAR values compared to large Fe_3_O_4_ MNPs. Upon immobilization in PEG1000, the H_cHyp_ of large Fe_3_O_4_ MNPs increased from 18 kA/m in water to 27 kA/m and 26 kA/m in liquid and soft solid PEG1000, respectively (Table 2). A similar increase of H_cHyp_ was recorded for small Fe_3_O_4_ MNPs dispersed in PEG1000. These behaviors suggest that H_cHyp_ is also influenced by the mobility of the MNPs and is not a parameter depending only on their intrinsic properties.

### 2.4. Cytotoxicity Assessment

In order to establish whether or not the MNPs are suitable for in vivo applications it is necessary first to evaluate their in vitro toxicity. These types of studies provide adequate information about the cytotoxicity of Fe_3_O_4_ MNPs and their cell internalization pathways.

The standard MTT assay was performed on four cell lines with different concentrations of Fe_3_O_4_ MNPs within the 0.05–0.2 mg/mL range. The cultured cells were incubated for 24 h at 37 °C in the same conditions. Upon incubation the cells did not show any sign of cell suffering; they were still confluent in the culture flask and were not detached, as observed under an inversed optical microscope. The results of the MTT assay are presented in Table S1. The small Fe_3_O_4_ MNPs did not show any cytotoxicity at 0.05 mg/mL. When the Fe_3_O_4_ MNPs concentration was increased up to 0.2 mg/mL, a cell viability of 94%–96% was obtained for all four types of cells. These results suggest that small Fe_3_O_4_ MNPs exhibit a negligible cytotoxicity profile after 24 h incubation time, in agreement with other similar Fe_3_O_4_ MNPs, incubated under the same concentrations [38]. At the lowest MNPs concentration of 0.05 mg/mL, large Fe_3_O_4_ MNPs do not exhibit any cytotoxicity. The cellular viability of all types of cells started to decrease once the MNPs concentration was increased. The cancer cells exhibited a cellular viability of 90%, while for the normal cells the cellular viability dropped to 80%. These results show a slightly higher toxicity of the large MNPs as compared to the small ones. Most of the nanotoxicity studies conducted on iron oxide MNPs have concluded that the toxicity mechanisms involved in the cell death are related to the production of reactive oxygen species (ROS) [21]. Although the cells and living systems possess different mechanisms to defend themselves against ROS, when this defense system is overwhelmed, the excess ROS can lead to lipid peroxidation, DNA strand breaks, alterations in gene transcription, generation of protein radicals and finally to cell death. One might consider, from a pure geometrical point of view, that for the same amount of magnetite, smaller NPs, having a much larger surface area, as compared to the larger ones, are able to generate more ROS and, therefore, are more toxic. However, a very large number of nanotoxicology studies have found out that many other parameters like, shape, size, chemical nature and especially surface coating of MNPs as well as the cell line type influence their toxicity. It was particularly demonstrated that PEG coating can significantly reduce the toxicity of MNPs and the effect is more pronounced for larger molecular mass polymer coatings [24,39]. In our case the small MNPs were synthesized in PEG 200, but the molecule was faintly visible at the surface of the MNPs as previously reported [20] and therefore we cannot, correlated to its low molecular mass, we cannot assert that the slightly lower toxicity of the smaller MNPs was due only to the presence of PEG 200 on their surface. 

### 2.5. Cell uptake Properties

**Figure 4:**
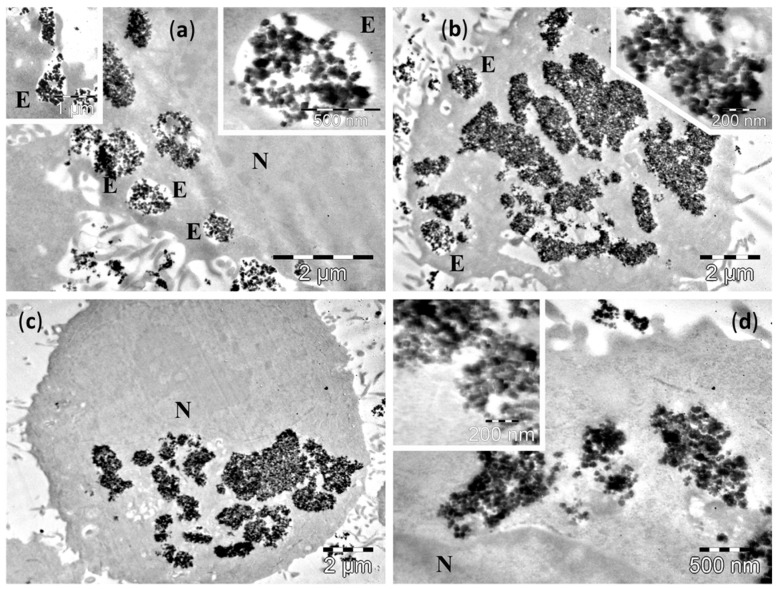
TEM images of D407 cells containing small Fe_3_O_4_ MNPs after 4 h (**a**,**b**) and 24 h (**c**,**d**) incubation time. The letters N denote the nucleus, whereas the letters E indicate the endosomes. Insets show: (**a**, left) formation of an endosome with small Fe_3_O_4_ MNPs; (**a**, right) detailed view of a Fe_3_O_4_ MNPs containing endosome; (**b**,**d**) detailed views of Fe_3_O_4_ MNPs in direct contact with the cytosol.

**Figure 5:**
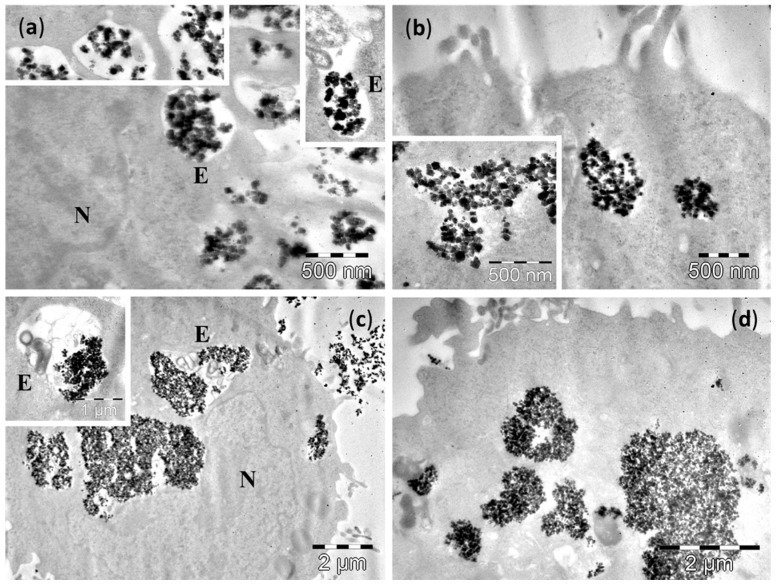
TEM images of A549 cells containing small Fe_3_O_4_ MNPs after 4 h (**a**,**b**) and 24 h (**c**,**d**) incubation. The letters N denote the nucleus, whereas the letters E indicate the endosomes. Insets show: (**a**, left) small Fe_3_O_4_ MNPs attached to plasma membrane prior to formation of endosomes; (**a**, right) formation of an endosome with small Fe_3_O_4_ MNPs; (**b**) detailed views of small Fe_3_O_4_ MNPs in direct contact with the cytosol; (**c**) detailed view of an endosome containing small Fe_3_O_4_ MNPs.

**Figure 6:**
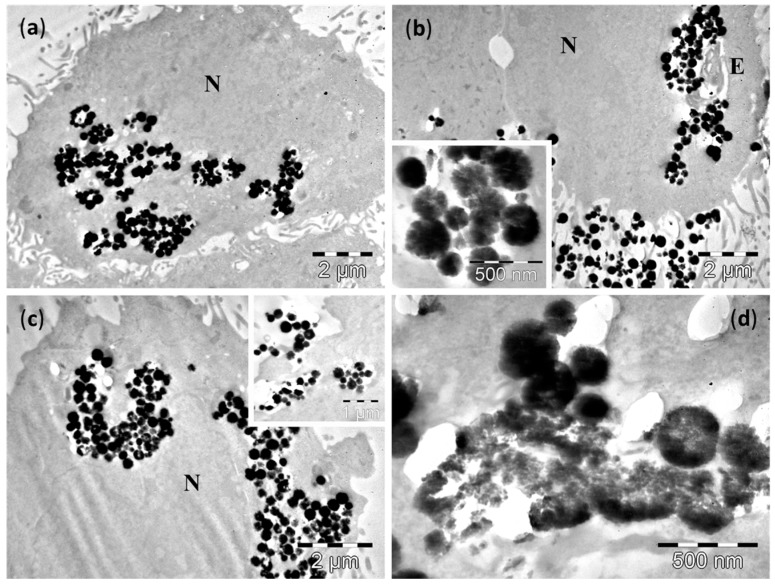
TEM images of D407 cells containing large Fe_3_O_4_ MNPs after 4 h (**a**,**b**) and 24 h of incubations (**c**,**d**). Many of the large Fe_3_O_4_ MNPs fragment into small Fe_3_O_4_ MNPs. The letters N denote the nucleus, whereas the letters E indicate the endosomes. Insets show: (**b**) detailed views of large Fe_3_O_4_ MNPs in direct contact with the cytosol; (**c**) formation of two endosomes with large Fe_3_O_4_ MNPs, and a cluster of MNPs in contact with the cytosol.

**Figure 7:**
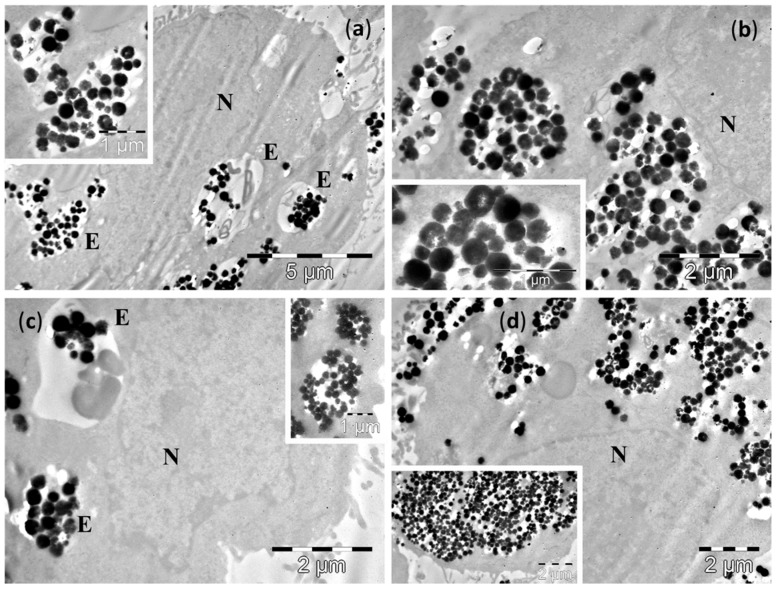
TEM images of A549 cells containing large Fe_3_O_4_ MNPs after incubations of 4 h (**a**,**b**) and 24 h (**c**,**d**). Many of the large Fe_3_O_4_ MNPs fragment into small Fe_3_O_4_ MNPs. The letters N denote the nucleus, whereas the letters E indicate the endosomes. Insets show: (**a**) a large Fe_3_O_4_ MNPs containing endosome; (**b**,**d**) detailed views of large Fe_3_O_4_ MNPs in direct contact with the cytosol; (**c**) a large Fe_3_O_4_ MNPs containing endosome, in the proximity of the plasma membrane, and next to two clusters of MNPs in contact with the cytosol.

TEM examination of cultured cells after 4 h incubation showed the presence of Fe_3_O_4_ MNPs inside the cytoplasm of normal cells, as well as of the malignant cells (upper panel of Figure 4, Figure 5, Figure 6 and Figure 7 and Figures S6–S9). After 24 h of incubation the number of NPs was much increased for all cell lines (lower panels of Figure 4, Figure 5, Figure 6 and Figure 7 and Figures S6–S9).

In the D407 cells, the small Fe_3_O_4_ MNPs were observed inside endosomal vesicles (Figure 4a), located in the cytoplasm, or even in the proximity of the nucleus. It is worth mentioning that many other small Fe_3_O_4_ MNPs were attached to the outer surface of plasma membrane, and the process of small Fe_3_O_4_ MNPs endocytosis was in progress at the moment of fixation (left inset of Figure 4a). The Fe_3_O_4_ MNPs were also observed in direct contact with the cytosol, after the disassembling of the endosomal vesicles (Figure 4b). In some cells they occupied large areas of the cytoplasm, and were still grouped in clusters. 

For 24 h incubation time, a high number of small Fe_3_O_4_ MNPs was found in the whole cytoplasm, but we could not identify them packed in vesicles, all the small Fe_3_O_4_ MNPs being in direct contact with the cytosol (Figure 4c,d). Despite the fact that in the cellular environment some small Fe_3_O_4_ MNPs were present, no Fe_3_O_4_ MNPs were attached on the plasma membranes of cells.

For the three cancer cells lines, A549 (Figure 5), MV35 (Figure S7) B16F10 (Figure S8), the cellular behaviour after the exposure to small Fe_3_O_4_ MNPs followed a common pattern, but with certain differences as compared to the normal D407 epithelial cells. After 4 h incubation, the Fe_3_O_4_ MNPs were observed mainly as aggregates more or less compact, in contact with the cytosol (upper panels of Figure 5, Figure S7 and S8). Nevertheless, cells containing Fe_3_O_4_ MNPs packed in endosomal vesicles were also observed, with the mention that the vesicles were localized near the plasma membrane. At 24 h, most cells had accumulated high amounts of Fe_3_O_4_ MNPs in the cytosol as membrane-free aggregates (upper panels of Figure 5, Figure S7 and S8), in many cases even in the proximity of nucleus. However, Fe_3_O_4_ MNPs-containing endosomes were still found in all three lines of cancer cells. Such vesicles were located deeper in the cytoplasm, and next to the plasma membrane (lower panels of Figure 5, Figure S7 and S8).

The relative high concentration of small Fe_3_O_4_ MNPs tested, as well as the high protein concentration of the DMEM culture medium, resulted in the internalization of important amounts of small Fe_3_O_4_ MNPs in all four cell types, as was revealed by TEM. The proteins contained in the culture medium facilitated the uptake of small Fe_3_O_4_ MNPs by their adsorption to the MNPs surface. The small Fe_3_O_4_ MNPs were found in cells even after a short incubation time. However, their cellular concentration was much increased for 24 h incubation time. Previous reports showed a time-dependent endocytosis [40,41], regardless of the ligands used to coat the MNPs [41]. On the other hand, it was demonstrated that DMEM had the ability to form a more abundant and stable protein coating at the nanoparticle surface as compared to other media [42]. The observation of endosomes containing small Fe_3_O_4_ MNPs in cancer cells after the long incubation indicated a continuous uptake process for a long time, thus making them suitable for therapeutic purpose. 

After 4 h incubation with large Fe_3_O_4_ MNPs, TEM examination of cultured cells also showed the presence of these large Fe_3_O_4_ MNPs in both categories of cells—normal and malignant (upper panels of Figure 6 and Figure 7, Figures S9 and S10). Similarly to the small Fe_3_O_4_ MNPs, the amount of large Fe_3_O_4_ MNPs in the cells was higher in all studied cell lines after the 24 h incubation (lower panels of Figure 6 and Figure 7, Figures S9 and S10).

Many large Fe_3_O_4_ MNPs were observed in the normal epithelial cells (D407 line) at 4 h (Figure 6a,b). In most of the examined cells, the large Fe_3_O_4_ MNPs were grouped in large clusters distributed in all the cytoplasm, between the plasma membrane of the cells and the nuclear envelope, in direct contact with the cytosol (Figure 6a,b). Very rare endosomal vesicles containing the large Fe_3_O_4_ MNPs were found in these cells (Figure 6b), and the membrane of such endosomes was discontinued (Figure 6b). In some of the cells, other large Fe_3_O_4_ MNPs were attached at the outer surface of plasma membrane, the process of MNPs endocytosis being in progress (Figure 6b). After 24 h, a higher amount of large Fe_3_O_4_ MNPs was observed in the cells (Figure 6c,d). They were all in direct contact with the cytosol, and—as in the case of small Fe_3_O_4_ MNPs—we could not identify large Fe_3_O_4_ MNPs packed in vesicles (Figure 6c,d), despite the fact that many of these clusters were found in immediate proximity of the plasma membrane and the endocytosis process seemed to continue (Figure 6c). A very interesting feature was noticed in the case of large Fe_3_O_4_ MNPs related to their biodegradation. As one can easily notice in the enlarged picture of Figure 6d, some of the large Fe_3_O_4_ MNPs within the cells and in contact with the cytosol are fragmented. On the other hand, other intact large Fe_3_O_4_ MNPs can also be seen near the fragmented ones. This interesting observation that some of large Fe_3_O_4_ MNPs are attacked and degraded and some are not, could indicate an all-or-nothing mechanism, which was very recently reported by Mazuel et al. [43] using a single endosome model. It seems that as the large Fe_3_O_4_ MNPs are attacked by inner cell molecules, once the coating of the large Fe_3_O_4_ MNPs was penetrated, a rapid degradation occurs, while other large Fe_3_O_4_ MNPs resist, due to their intact coatings. The same behavior was noticed in intact cells as can be seen in Figures S9 and S10.

In the three cancer cells lines, A549 (Figure 7), MV35 (Figure S9) and B16F10 (Figure S10), the endocytosis process displayed some particularities after the exposure to large Fe_3_O_4_ MNPs. The first two lines had a similar behavior to the normal cells, while for the third line a difference was recorded.

After 4 h of incubation, the large Fe_3_O_4_ MNPs were observed in both the A549 cells and MV35 cells inside endosomes with various distribution within the cytoplasm, and as free aggregates, more or less compact, in contact with the cytosol (Figure 7a,b and Figure S9a,b). In the B16F10 line, no membrane surrounding large Fe_3_O_4_ MNPs was found (Figure S10a,b), even though some of the large Fe_3_O_4_ MNPs aggregates resembled endosomes (Figure S10b). Many of the large Fe_3_O_4_ MNPs located either in the cytosol (Figure 7b and Figure S10b) or in the endosomes (Figure S9b) started to fragment into smaller pieces. At 24 h, most of the cells in the A549 and MV35 lines showed large Fe_3_O_4_ MNPs containing endosomes in the proximity of nucleus (Figure 7c), or next to the plasma membrane (Figure 7c). No endosomes were found in the B16F10 cells (Figure S9c,d). On the other hand, the large Fe_3_O_4_ MNPs accumulated in high amounts in the cytosol as membrane-free aggregates (Figure 7c,d, Figures S9c,d and S10c,d), sometimes even in very high amounts (Figures S9d and S10d).

As compared to the small Fe_3_O_4_ MNPs, the larger ones were released from the endosomes into the cytosol faster. The large Fe_3_O_4_ MNPs release was probably achieved due to their larger sizes, and by a mechanical mechanism involving the breaking of the endosomal membrane apart. We believe this aspect could influence their biological properties by increasing their ability to interact with the cellular systems. The large Fe_3_O_4_ MNPs fragmentation represent an important finding that could be responsible for the enhancement of their biological effects, however the mechanisms of large Fe_3_O_4_ MNPs fragmentation, as well as its relevance for the effects of these large Fe_3_O_4_ MNPs, remain to be solved by further studies.

There is increased interest in the study of the various toxic effects of nanoparticles due to the fact that humans and animals are exposed to various nanoscale materials as more and more consumer products claim to include nanoparticles and the new emerging field of nanotechnology has become another threat to human life. In a recent report [44] it is mentioned that in 2014 they were 1814 nanoparticle-based consumer products and this figure is continuously growing with many of these products not being well characterized from their NP composition, size and toxicological effects points of view. Recent reviews concerning the toxicological effects of various nanomaterials [23] have emphasized that there are limited and, sometimes conflicting data about the toxicity of nanoparticles and how their size, surface area, concentration can be controlled in order to optimize and limit the nanoparticles’ cellular toxicity. A common belief is that nanoparticle sized materials are more toxic than the bulk materials and is usually suggested that toxicities are inversely proportional to the size of the nanoparticles. In the particular case of MNPs their toxic effect depends on the structural properties of MNPs, dosage, solubility, surface chemistry, coating, biodegradation, biodistribution [22,45]. From literature data it seems that the surface coating of MNPs is one of the most relevant parameter related to toxicity. Our data are consistent with previous reports which showed that uncoated MNPs exhibit cytotoxicity above a certain concentration level, usually 100 μg/mL [46].

## 3. Materials and Methods

### 3.1. Synthesis Method

All the reagents employed in this study were of analytical grade and were used without any further purification. The synthesis of magnetic nanoparticles was performed with the following products: iron(III) chloride hexahydrate (FeCl_3_·6H_2_O, ≥98%, Roth, Karlsruhe, Germany), ethylene glycol (EG, ≥ 99%, Roth), polyethylene glycol 200 (PEG 200, ≥99%, Roth) and sodium acetate trihydrate (NaOAc, ≥ 99.5%, Roth). The synthetic procedure for the preparation of Fe_3_O_4_ was as follows: FeCl_3_·6H_2_O (0.335 g) and NaOAc (1.8 g) were mixed and dissolved in PEG 200 (60 mL) or FeCl_3_·6H_2_O (0.67 g) and NaOAc (1.8 g) were mixed and dissolved in EG (40 mL). The solutions were stirred thoroughly at room temperature for 30 min, transferred in glass and sealed in a vessel made of stainless steel and heated at 240 °C (PEG200) and 200 °C (EG) for 6 h. The final temperature was reached at heating rates of 3 °C/min. The vessel was cooled at room temperature, the excess liquid was discharged and the obtained black precipitates were washed with double distilled water, several times, in order to remove the excess of ligands and unreacted precursors. Finally, the black precipitate was dispersed and kept in 10 mL of double distilled water for further analysis.

### 3.2. Experimental Methods

TEM images of magnetic nanoparticles were taken on a Hitachi HT7700 (Hitachi Ltd. Tokyo, Japan) equipped with an 8 megapixel CCD camera and operating at 100 kV in high contrast mode. For TEM examination, 5 μL drop of nanoparticle suspension was deposited on carbon-coated copper grids. After 2 min the excess liquid was removed by filter paper and the sample was left to dry under ambient air.

X-ray diffraction (XRD) measurements were carried out on powder samples at room temperature on a Bruker D8 Advance diffractometer (Bruker-AXS GmbH, Karlsruhe, Germany) using Cu Kα radiation. The lattice parameters and phase percentages were calculated using the free FullProf software (http://www.crystalimpact.com/match/download.htm).

Dynamic light scattering (DLS) measurements were taken using a Zetasizer Nano ZS90 (Malvern Instruments, Worcestershire, UK) in a 90° configuration. Three cycles of 10 measurements, 5 s each, were performed for each sample. Measurements were performed for sample concentrations in the range 1 mg/mL-1 μg/mL. After each dilution the samples were sonicated for at least 30 s.

Magnetic measurements were performed on powder samples in the 4–300 K temperature range in external applied fields up to 5 T, using a vibrating sample magnetometer (VSM) produced by Cryogenic Limited (London, UK).

Hyperthermia measurements were recorded with a magnetic heating system Easy Heat 0224 provided by Ambrell (Scottsville, NY, USA). The samples, usually 0.5 mL of Fe_3_O_4_ MNPs suspensions at different concentrations were placed in a thermally insulated vial, at the center of an 8 turn coil, connected to the remote heat station of the device. With this setup, alternating magnetic fields with strengths between 5 kA/m and 65 kA/m at a frequency of 355 kHz were generated in the center of the coil. The temperature was measured using a fiber-optic probe, placed in the center of the vial, connected to a computer, providing the temperature values each second.

### 3.3. Cell Lines

Four type of cell lines we used in our study, 1 normal cell line and 3 cancer cell lines. The human melanoma cell line MW35 were cultured in RPMI 1640 medium supplemented with 10% fetal bovine serum (HyClone Lab, Inc. Logan, UT, USA), 1 mM glutamine (Sigma-Aldrich, St. Louis, MO, USA), 1% antibiotic-antimycotic 100× (Sigma-Aldrich). The mouse melanoma cell line B16F10 was maintained in DMEM (Sigma-Aldrich) medium supplemented with 10% fetal bovine serum (HyClone Lab, Inc.), 1 mM glutamine (Sigma-Aldrich), 1% antibiotic antimycotic 100× (Sigma-Aldrich). Human lung adenocarcinoma A549 cells were grown in RPMI 1640 (Sigma-Aldrich) medium with 1% Antibiotic-antimycotic 100× (Sigma-Aldrich) and 10% FBS (Hyclone). Human retinal pigment epithelial D407 cells were cultured in DMEM high glucose (Sigma-Aldrich) medium supplemented with 10% fetal bovine serum (Hyclone), 1 mM glutamine (Sigma-Aldrich), 1% antibiotic-antimycotic 100× (Sigma-Aldrich). Cultures were maintained at 37 °C in 5% CO_2_ and 95% relative humidity.

### 3.4. Cytotoxicity Assays

For the cell survival the cell lines were plated (1 × 10^5^ cells/well) in 96-well plates for 24 h in normal propagation media. The culture medium was then replaced with complete medium containing six types of nanoparticles in 3 different concentrations (0.2 mg/mL, 0.1 mg/mL, and 0.05 mg/mL). The negative controls were represented by cells lines cultivated without nanoparticles in normal expansion medium. Each experiment was carried out in triplicate and behaviour of the cells was evaluated using an inversed optical microscope (Nikon TS100, Nikon Instruments, Wien, Austria). After 24 h of exposure the number of viable cells was investigated with the MTT (3-(4,5-dimethylthiazol-2-yl)-2,5-diphenyl tetrazolium bromide (Sigma-Aldrich) assay. Therefore, 100 µL MTT solution (5 mg/mL) were added in each well and incubated for 1 h al 37 °C. The formazan particles were solubilized with dimethyl sulfoxide (DMSO) (Sigma-Aldrich). The absorbance was read at 550 nm using a microplate reader (Bio-Rad, Hercules, CA, USA). The results were calculated as survival percent compared with untreated control.

### 3.5. Cellular Uptake

Immediately after the incubation with the Fe_3_O_4_ MNPs having a concentration of 0.2 mg/mL (after 4 h and 24 h respectively) the cells were processed for TEM. They were prefixed directly in the culture flask with 2.7% glutaraldehyde (Electron Microscopy Sciences, Hatfield, PA, USA) in 0.1 M phosphate buffer (pH 7.4) at 4 °C for 1.5 h. After a centrifugation at 1500 RPM for 10 min the cells were washed four times with 0.1 M phosphate buffer (pH 7.4), and then post-fixed for 1.5 h with 1.5% osmium tetroxide (Sigma-Aldrich) at 4 °C. They were next dehydrated in acetone series (30% to 100%), infiltrated and embedded in Epon 812 resin (Fluka, Buchs, Switzerland). The blocks polymerized for 72 h at 60 °C were trimmed and cut with glass knives on a Bromma 8800 ULTRATOME III (LKB, Stockholm, Sweden). The ultrathin sections (60–80 nm) were collected on 3 mm copper grids (with formvar film), and contrasted for 7 min with uranyl acetate (Merck, Billerica, MA, USA). The samples were examined on the JEOL-JEM 1010 transmission electron microscope (Jeol Ltd., Tokyo, Japan), equipped with a Mega VIEW III camera (Olympus, Soft Imaging System, Münster, Germany) and operating at 80 kV.

## 4. Conclusions

Two classes of Fe_3_O_4_ MNPs were synthesized by using a polyol based method and systematically investigated and compared for their structural, magnetic, hyperthermic, cytotoxic and cell uptake properties. While PEG 200 favors the formation of Fe_3_O_4_ MNPs with cubic, polyhedral and octahedral shapes with an average size of ~34 nm, within similar temperature and concentration conditions, EG produces large, spherical Fe_3_O_4_ MNPs with an average size of ~270 nm. XRD studies showed that both samples consisted of pure magnetite, with almost similar crystallite size, with small Fe_3_O_4_ MNPs being mostly single crystals and with a polycrystalline structure in the case of large Fe_3_O_4_ MNPs. Large differences were measured in the hyperthermia properties of the Fe_3_O_4_ MNPs. For both classes of Fe_3_O_4_ MNPs a sigmoidal dependence of the SAR = f(H) curves were detected. The experimental data, were very well fitted with a simple logistic function. Three-times higher SAR_max_ values were obtained for the single crystal Fe_3_O_4_ MNPs. The lower values of the SAR_max_ in the case of large Fe_3_O_4_ MNPs were attributed to dipolar magnetic interactions between the crystallites within large MNPs, our results clearly emphasizing the role of monocrystallinity in achieving high heating capabilities for MNPs. A slight increase of the SAR values was noticed when the hyperthermia experiments started at 37 °C as compared to those starting at room temperature (25 °C) and no significant changes were recorded when the Fe_3_O_4_ MNPs were suspended in cell culture medium mimicking the outer cell compartment. In a highly viscous medium (liquid PEG 1000) or in a soft solid (PEG 1000 below the melting point) a dramatic decrease of SAR for both types of MNPs was measured. The small Fe_3_O_4_ MNPs showed a double SAR_max_ value as compared to large Fe_3_O_4_ MNPs. Our results clearly demonstrate that the polyol based method can be successfully used for the synthesis of high crystallinity Fe_3_O_4_ MNPs with remarkable high SAR values, due to the high temperatures used. We strongly believe that by refining this method one can finely tune both the size and the dispersity of MNPs leading to significantly improved hyperthermic properties. 

The MTT assays performed on four cell lines revealed almost no toxicity for the small Fe_3_O_4_ MNPs and a very small level of toxicity for large Fe_3_O_4_ MNPs, in accordance with other published data for the concentration range used (up to 0.2 mg/mL). The cellular uptake experiments showed that both types of MNPs penetrated the cells through endocytosis, in a time dependent manner. After 24 h of incubation the Fe_3_O_4_ MNPs were released from the endosomes and got in direct contact with the cytosol. The large Fe_3_O_4_ MNPs presented a faster release as compared to the small Fe_3_O_4_ MNPs ones, probably due to their larger sizes The large Fe_3_O_4_ MNPs fragmentation through an all-or-nothing mechanism represent an important finding as well. The mechanism responsible of large Fe_3_O_4_ MNPs fragmentation as well as its biological relevance remain to be solved by further studies.

## Figures and Tables

**Table 1:** Magnetic properties of Fe_3_O_4_ MNPs.

Sample	4 K				300 K			
	M_s_ (emu/g)	H_c_ (kA/m)	M_r_ (emu/g)	K_eff_ (kJ/m^3^)	M_s_ (emu/g)	H_c_ (kA/m)	M_r_ (emu/g)	K_eff_ (kJ/m^3^)
Small-Fe_3_O_4_	82	20	16	11.2	68	2.5	2	1.16
Large-Fe_3_O_4_	75	27	21	13.8	68	6	7	2.79

**Table 2:** Fitting results of SAR evolution with AC magnetic field amplitude.

Sample	Conditions	C (mg/mL)	SAR_max_ (W/g)	H_cHyp_ (kA/m)	Power Coefficient n
Small Fe_3_O_4_	Water—25 °C	4	1352	19	5.7
	Water—25 °C	2	1304	19	6.3
	Water—37 °C	4	1442	19	5
	Cell culture medium—37 °C	4	1348	20	5.6
	PEG1K—25 °C	4	179	27	2
	PEG1K—37 °C	4	522	26	4.4
Large Fe_3_O_4_	Water—25 °C	4	370	18	3.1
	Water—25 °C	2	363	18	3.1
	Water—37 °C	4	431	18	3.1
	Cell culture medium—37 °C	4	430	22	2.9
	PEG1K—25 °C	4	95	27	1.81
	PEG1K—37 °C	4	270	29	2.75

## Supplementary Materials

Supplementary materials can be accessed at: http://www.mdpi.com/1420-3049/21/10/1357/s1.
