# Supplementary material for: Small versus Large Iron Oxide Magnetic Nanoparticles: Hyperthermia and Cell Uptake Properties
Source: Molecules. 2016 Oct 13;21(10):1357. doi: 10.3390/molecules21101357 (PMC6274490; doi:10.3390/molecules21101357)
Supplement: Supplementary file 1 [file molecules-21-01357-s001.pdf]

# Supplementary Materials: Small Versus Large Iron Oxide Magnetic Nanoparticles: Hyperthermia and Cell Uptake Properties

**Cristian Iacovita, Adrian Florea, Roxana Dudric, Emoke Pall, Alin Iulian Moldovan, Romulus Tetean, Rares Stiufiuc and Constantin Mihai Lucaciu**

## 1. Specific Absorption Rate (SAR) Determination

Experimentally, the specific absorption rate (SAR), called sometimes specific power loss (SLP), is defined as the heat released from a suspension of magnetic nanoparticles (MNPs) in unit time reported to their mass, expressed usually in *W/g*:

$$\mathrm{SAR} = \frac{\mathrm{P}}{\mathrm{m_{MNP}}} = \frac{\mathrm{Q}}{\Delta\mathrm{t}\,\mathrm{m_{MNP}}}$$

the heat being calculated as the equivalent heat needed to increase the sample temperature with the same number of *K* in a given time interval,

$$\mathrm{Q} = \mathrm{m\,c}\,\Delta\mathrm{T}$$

where *Q* is the heat, *m* is the mass of the sample subjected to is heating in alternating magnetic field, *c* specific heat of the sample (with a good approximation one can consider specific heat of water for dilute magnetic nanoparticles suspended in water), ΔT is the temperature increase of the sample, and Δt is the time during which the alternating magnetic field was applied.

The MNPs were weighed and then were suspended in different media to the desired concentrations (4 mg/mL, 2 mg/mL) for measurements of their hyperthermia properties. An amount of 0.5 mL of each sample was placed in round bottom test tubes with a total 2 mL capacity which was then positioned in the center of the coil. In order to preserve a constant temperature environment inside coil the text tubes were surrounded by a low diameter tubing through which water has been circulated at the desired temperature. Care was taken for the samples to be placed in the same position each time inside the coil to be subject to the same field strengths. The temperature was measured with an optical fiber thermocouple connected to a computer which allows temperature measurement every second. Note that fiber optics thermocouple provides precise temperature measurement without being influenced by the magnetic field. Every time the optical fiber thermocouple was placed in the middle of the volume occupied by the aqueous solution of magnetic nanoparticles. All samples were measured at least 3 times and the samples were sonicated for 30 s before the measurement in order to ensure uniform dispersion of the particles in the suspension medium. Since the hyperthermia measurements were performed in a non-adiabatic environment, we have taken into account only the linear portion of the time dependence of the temperature (see black rectangles in both Figures S2 and S3), for the SAR determination. In the SAR calculation we have considered for water and cell culture medium: ϱ = 1 g/mL, c = 4186 J/kgK; for PEG1K: ϱ = 1.03 g/mL, c = 2100 J/kgK. Magnetic field calibration was performed as described in ref. [1].

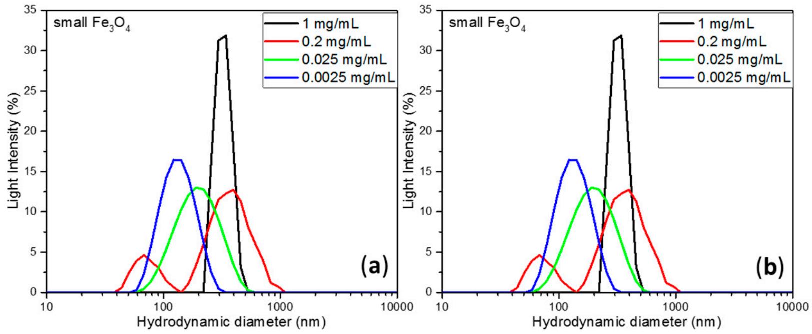


**Figure S1.** Hydrodynamic diameter of small (**a**) and large (**b**) $Fe_3O_4$ MNPs as a function of the concentration.

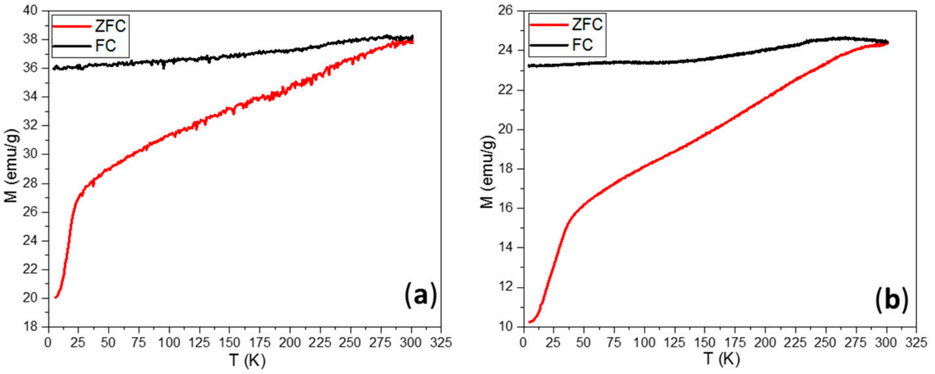


**Figure S2.** ZFC and FC magnetization curves of (**a**) small and (**b**) large $Fe_3O_4$ MNPs, acquired in an external magnetic field of 50 mT.

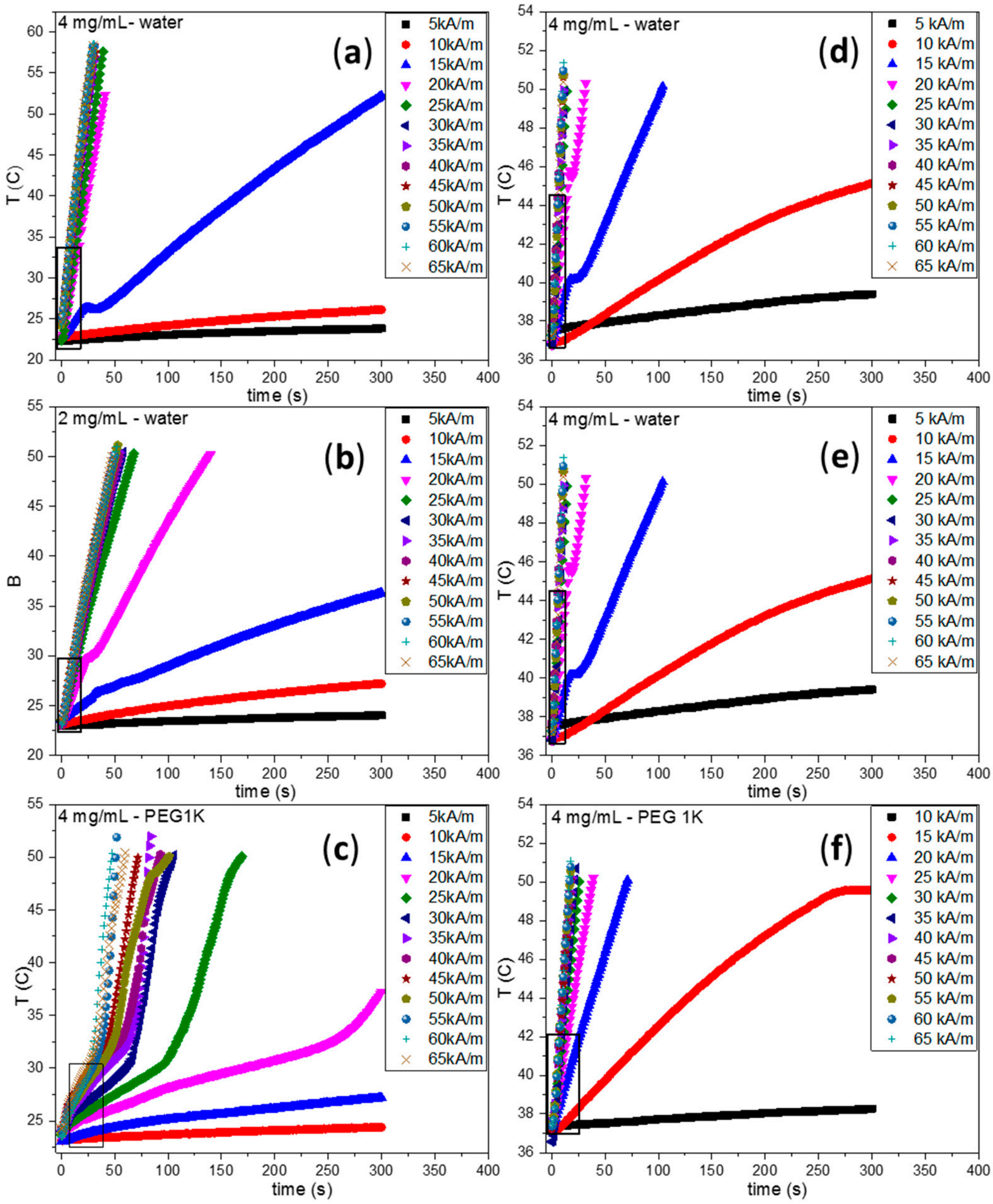


**Figure S3.** Heating curves of small $Fe_3O_4$ MNPs dispersed in different media and recorded starting with room temperatures (left panels: **a**–**c**) and 37 °C (right panels: **d**–**f**) as a function of AC magnetic field amplitudes at 355 kHz. The black rectangles frame the slope ($\Delta T/\Delta t$) used in SAR determination.

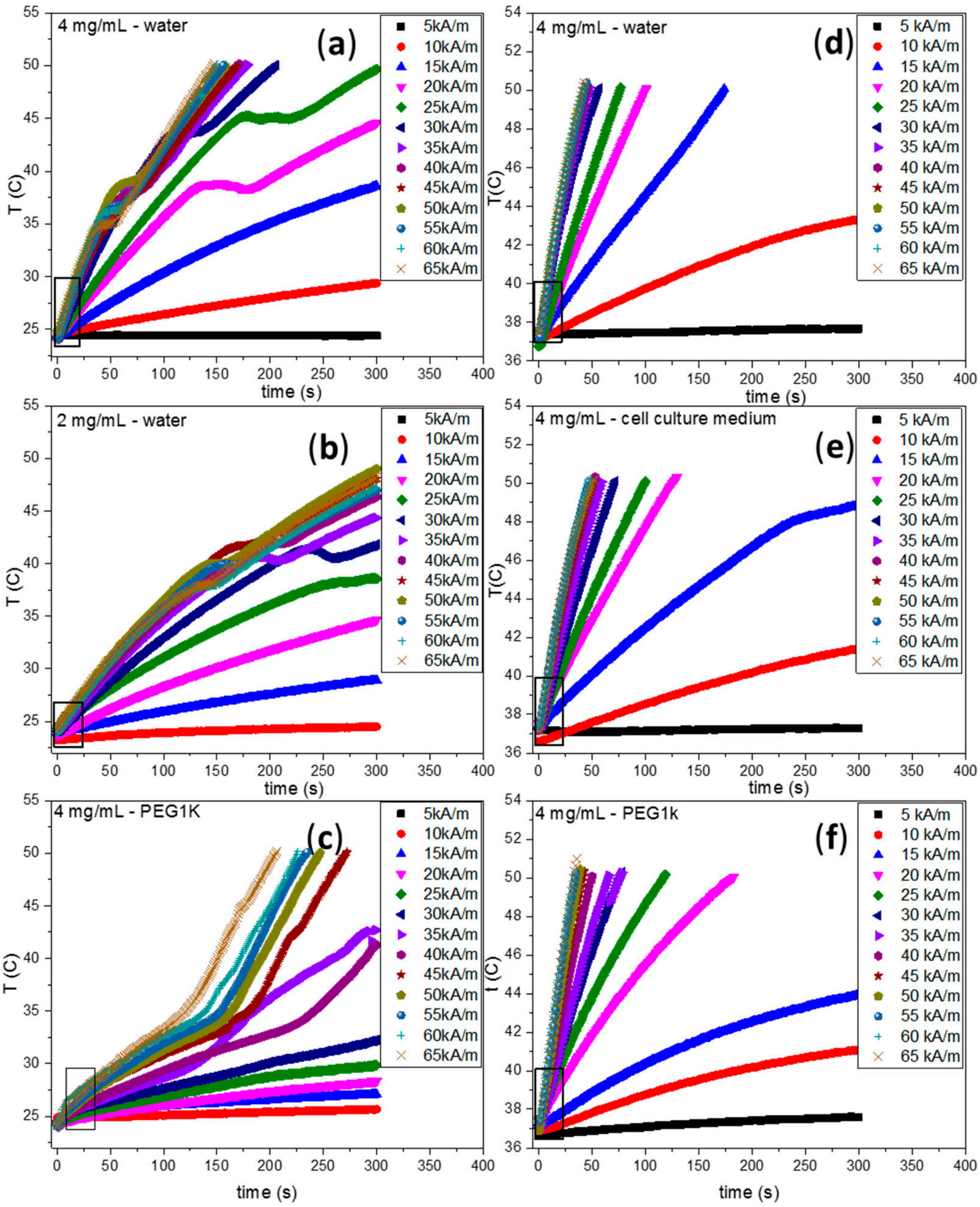


**Figure S4.** Heating curves of large $Fe_3O_4$ MNPs dispersed in different media and recorded starting with room temperatures (left panels: **a**–**c**) and 37 °C (right panels: **d**–**f**) as a function of AC magnetic field amplitudes at 355 kHz. The black rectangles frame the slope (ΔT/Δt) used in SAR determination.

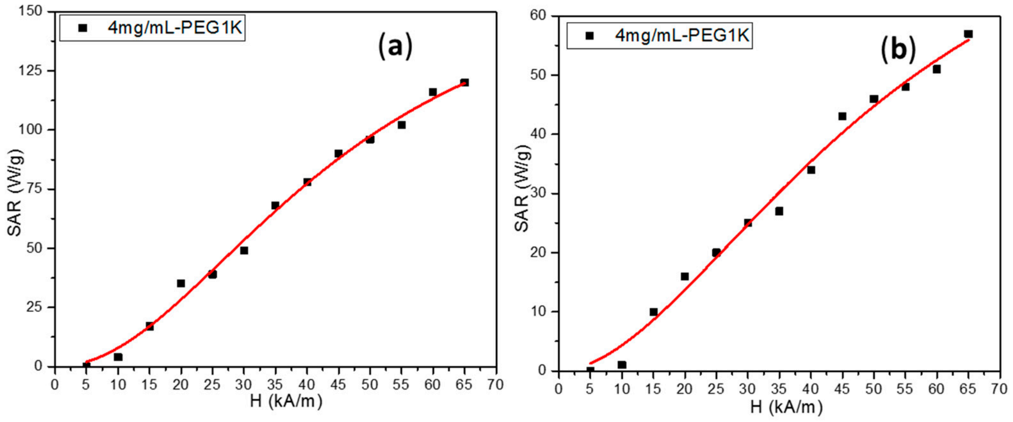


**Figure S5.** SAR values of $Fe_3O_4$ MNPs synthesized in (**a**) PEG 200 and (**b**) EG as a function of AC field amplitudes and at a frequency of 355 kHz. The $Fe_3O_4$ MNPs are dispersed in soft solid PEG1000 and the hyperthermia measurements have been performed starting with room temperature up to 37 °C. The data are fitted with a sigmoidal function (red lines).

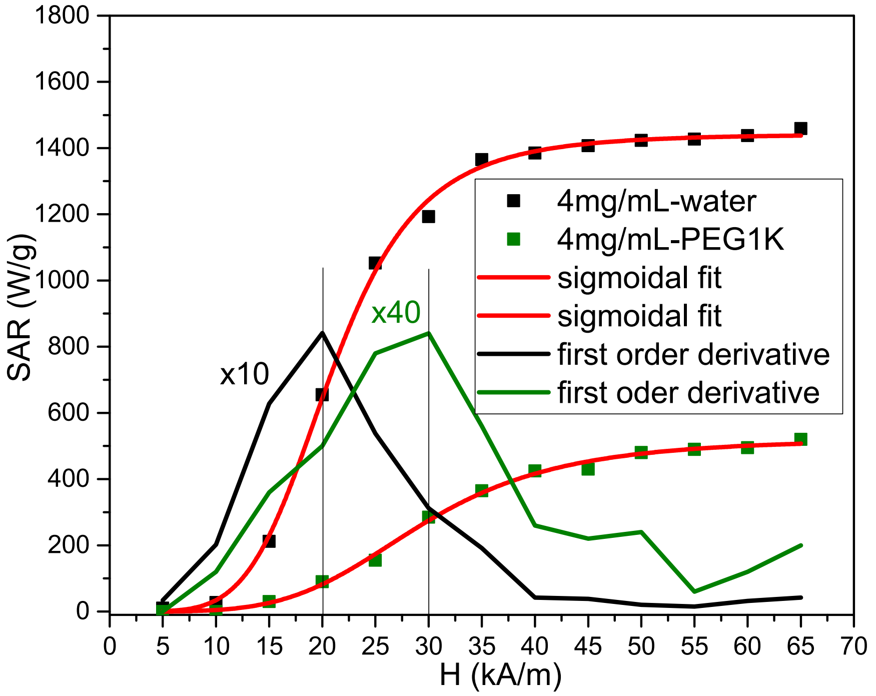


**Figure S6.** The SAR values of small $Fe_3O_4$ MNPs as a function of AC field amplitudes in water and PEG1K. The experimental results have been fitted with a sigmoidal function (red lines). The first-order derivatives of the experimental data were multiplied by 10 and 40 for clarity (black and green lines). The maxima in first-order derivative correspond to the inflection points of sigmoidal function and give the values of $H_{cHyp}$.

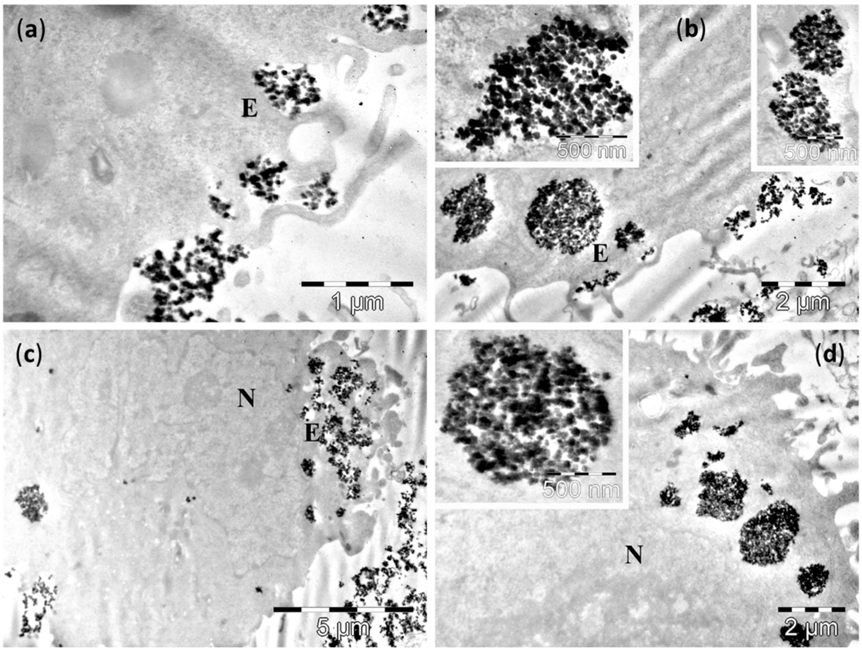


**Figure S7.** TEM images of MV35 cells containing small $Fe_3O_4$ MNPs after incubation of 4 h (**a** and **b**) and 24 h (**c** and **d**). The letters N denote the nucleus, whereas the letters E indicate the endosomes. Insets show detailed views of small $Fe_3O_4$ MNPs in direct contact with the cytosol: still grouped (**b**, right and **d**) and beginning to disperse (**b**, left).

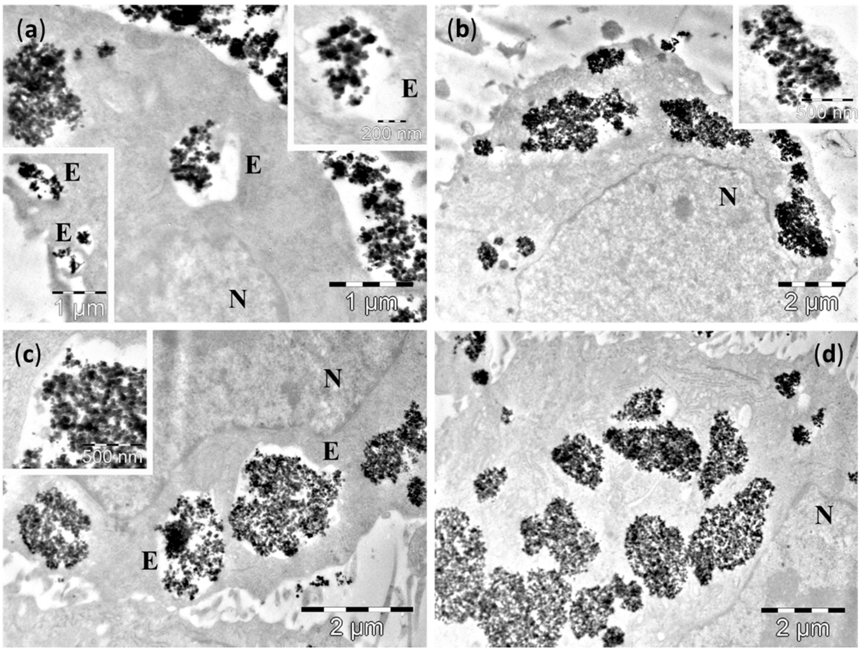


**Figure S8.** TEM images of B16F10 cells containing small $Fe_3O_4$ MNPs after incubation of 4 h (**a** and **b**) and 24 h (**c** and **d**). The letters N denote the nucleus, whereas the letters E indicate the endosomes. Insets show: (**a**, left) formation of an endosome with small $Fe_3O_4$ MNPs and some newly formed endosomes; (**a**, right) detailed view of a small $Fe_3O_4$ MNPs containing endosome; (**b**) detailed view of small $Fe_3O_4$ MNPs in direct contact with the cytosol; (**c**) detailed view of the largest endosome containing small $Fe_3O_4$ MNPs from the main image.

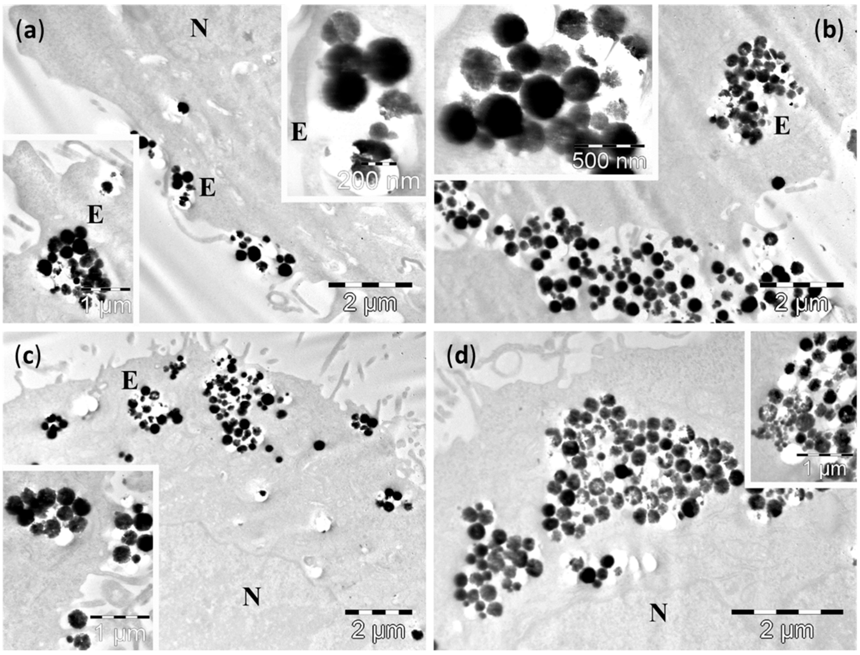


**Figure S9.** TEM images of MV35 cells containing large $Fe_3O_4$ MNPs after incubations of 4 h (**a** and **b**) and 24 h (**c** and **d**). Many of the large $Fe_3O_4$ MNPs fragment into small $Fe_3O_4$ MNPs. The letters N denote the nucleus, whereas the letters E indicate the endosomes. Insets show: (**a**) detailed views of large $Fe_3O_4$ MNPs containing endosomes; (**b**–**d**) detailed views of large $Fe_3O_4$ in direct contact with the cytosol.

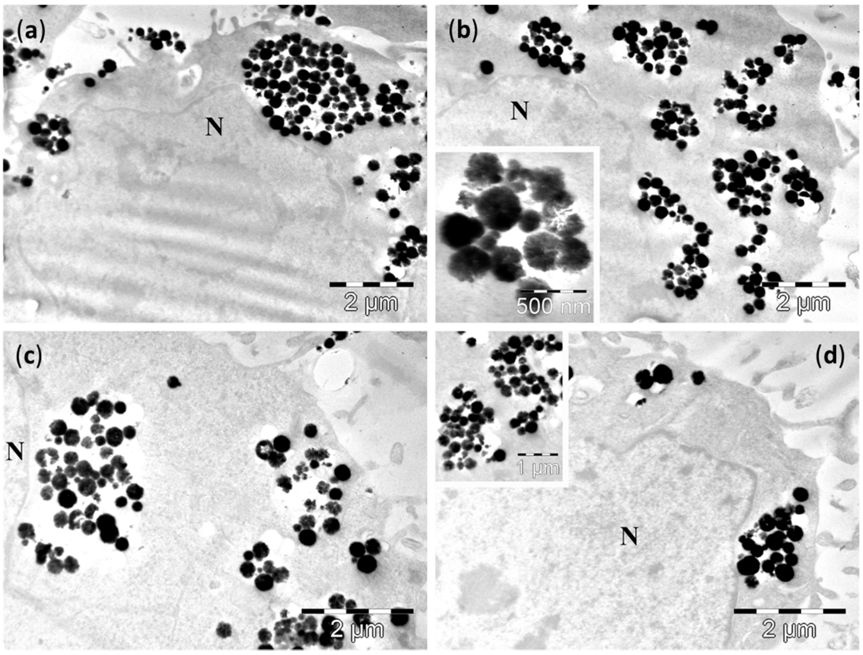


**Figure S10.** TEM images of B16F10 cells containing large $Fe_3O_4$ MNPs after incubations of 4 h (**a** and **b**) and 24 h(**c** and **d**). Many of the large $Fe_3O_4$ MNPs fragment into small $Fe_3O_4$ MNPs. The letters N denote the nucleus, whereas the letters E indicate the endosomes. Insets show detailed views of large $Fe_3O_4$ MNPs in direct contact with the cytosol.

**Table S1.** Cell viabilities exhibited by the four types of cells upon 24 h incubation with both types of $Fe_3O_4$ MNPs

| Cell lines | Small $Fe_3O_4$ MNPs Cell Viability | | | Large $Fe_3O_4$ MNPs Cell Viability | | |
|---|---|---|---|---|---|---|
| | **0.2 mg/mL** | **0.1 mg/mL** | **0.05 mg/mL** | **0.2 mg/mL** | **0.1 mg/mL** | **0.05 mg/mL** |
| D407 | 96% | 98% | 100% | 80% | 87% | 100% |
| A548 | 95% | 98% | 100% | 88% | 95% | 100% |
| B12F10 | 95% | 97% | 100% | 90% | 93% | 100% |
| MW35 | 94% | 98% | 100% | 89% | 92% | 100% |

## Reference

1. Iacovita, C.; Stiufiuc, R.; Radu, T.; Florea, A.; Stiufiuc, G.; Dutu, A.; Mican, S.; Tetean, R.; Lucaciu, C.M., Polyethylene glycol-mediated synthesis of cubic iron oxide nanoparticles with high heating power. *Nanoscale. Res. Lett.* **2015**, *10*, 1–16. doi: 10.1186/s11671-015-1091-0.
